# Opportunities and challenges of e-learning in vocational training in General Practice – a project report about implementing digital formats in the KWBW-Verbundweiterbildung^plus^

**DOI:** 10.3205/zma001390

**Published:** 2020-12-03

**Authors:** Christian Förster, Jessica Eismann-Schweimler, Sandra Stengel, Martina Bischoff, Monika Fuchs, Andreas Graf von Luckner, Thomas Ledig, Anne Barzel, Andy Maun, Stefanie Joos, Joachim Szecsenyi, Simon Schwill

**Affiliations:** 1 Universitätsklinikum Tübingen, Institut für Allgemeinmedizin und Interprofessionelle Versorgung, Tübingen, Germany; 2 Universitätsklinikum Freiburg, Lehrbereich Allgemeinmedizin, Freiburg, Germany; 3 Universitätsklinikum Heidelberg, Abteilung für Allgemeinmedizin und Versorgungsforschung, Heidelberg, Germany; 4 Universitätsklinikum Ulm, Institut für Allgemeinmedizin, Ulm, Germany

**Keywords:** postgraduate medical education, residency, vocational training, competence centers for postgraduate medical education, general practice, primary care, e-learning, webinar, blended learning

## Abstract

**Background: **The task of the Competence Centers for vocational training (KW) is to increase the attractiveness and quality of vocational (=post-graduate) training in general practice. For this purpose, they offer, among other things, a structured seminar program for post-graduate trainees in general practice (GP-trainees). During the Covid-19 pandemic the seminar program of the KWBW-Verbundweiterbildung^plus®^ in Baden-Württemberg was converted to digital formats. The goal of the paper is to evaluate the acceptance by the GP-trainees and lecturers, to describe experiences with the conversion to e-learning and to derive recommendations with regard to the future orientation of seminar programs in post-graduate as well as continuing medical education.

The implementation was based on a modified Kern-cycle and aimed at offering eight teaching units of 45 minutes each to a large number of GP-trainees. It tried to maintain the high quality of content and education as well as the interactive character of the previous seminars. For this purpose, the events were designed as synchronous webinars (six units) with asynchronous preparation and post-processing (two units) according to the flipped classroom method. The evaluation by the participating GP-trainees and lecturers was performed online using a multi-center developed and pre-piloted questionnaire.

**Results and discussion:** N=101 GP-trainees participated in the evaluation of five individual seminar days in the second quarter of 2020 (response rate 97%). 58% (N=59) of the trainees were satisfied or very satisfied with the implementation. 82% (n=83) rated pre-tasks as helpful. 99% (n=100) would participate in an online seminar again. For 52% (n=53) of the trainees, the attitude towards e-learning had changed positively. The main advantages mentioned were no travel, save in time and costs as well as increased flexibility. The main disadvantages mentioned were less personal interaction and technical obstacles.

The high acceptance of the new digital format showed the fundamental potential of e-learning in continuing medical education. The experiences can be a source of inspiration for other departments and KW. However, it also shows that important goals of KW, such as the personal interaction of the peer group, could not be achieved. In the future, it is important to develop a suitable mix of presence and digital formats with the aim to improve the attractiveness as well as sustainability of continuing medical education.

## Zusammenfassung

**Hintergrund: **Um die Attraktivität und Qualität der Weiterbildung in der Allgemeinmedizin zu steigern, bieten die Kompetenzzentren Weiterbildung (KW) u.a. ein strukturiertes Seminarprogramm für Ärztinnen und Ärzte in Weiterbildung (ÄiW) an. Im Zuge der Covid-19-Pandemie wurde das Seminarprogramm der KWBW-Verbundweiterbildung^plus®^ in Baden-Württemberg auf digitale Formate umgestellt. Ziel der Arbeit ist es, die Akzeptanz bei ÄiW und Dozierenden zu evaluieren, die Erfahrungen bei der Umstellung auf E-Learning zu beschreiben und daraus Empfehlungen abzuleiten im Hinblick auf die zukünftige Ausrichtung von Seminarprogrammen in der Facharztweiterbildung.

Die Umsetzung orientierte sich an einem modifizierten Kern-Zyklus und hatte zum Ziel, einer großen Zahl von ÄiW weiterhin acht Unterrichtseinheiten (UE) à 45 min anbieten zu können und dabei die hohe inhaltliche und didaktische Qualität sowie den interaktiven Charakter der bisherigen Seminare beizubehalten. Dazu wurden die Veranstaltungen als synchrone Webinare (sechs UE) mit asynchroner Vor- und Nachbereitung (zwei UE) im Sinne der *flipped classroom* Methode konzipiert. Die Evaluation durch die teilnehmenden ÄiW und Dozierenden erfolgte online mittels eines multizentrisch entwickelten und vorab pilotierten Fragebogens.

**Ergebnisse und Diskussion: **An der Evaluation der fünf Einzelseminartage im zweiten Quartal 2020 nahmen N=101 ÄiW teil (Rücklauf 97%). 58% (N=59) der ÄiW waren mit der Umsetzung zufrieden oder sehr zufrieden. 82% (N=83) empfanden die Vorbereitungsaufgaben als hilfreich. 99% (N=100) würden erneut an einem Online-Seminar teilnehmen. Bei 52% (N=53) der ÄiW hat sich die Einstellung gegenüber E-Learning positiv geändert. Vorteile ergäben sich v.a. durch den Wegfall der Anreise, Zeit- und Kostenersparnisse sowie eine erhöhte Flexibilität. Als nachteilig wurden insbesondere der geringere persönliche Austausch und technische Hürden genannt.

Die hohe Akzeptanz des neuen digitalen Formats zeigt das grundsätzliche Potenzial von E-Learning in der ärztlichen Weiterbildung. Die Erfahrungen können Impulsgeber für andere Fachbereiche und KW sein. Allerdings zeigt sich auch, dass wichtige Ziele der KW, wie der persönliche kollegiale Austausch, damit nicht erreicht werden können. Für die Zukunft gilt es, eine geeignete Mischung aus Präsenz und digitalen Formaten zu entwickeln mit dem Ziel, die ärztliche Weiterbildung noch attraktiver und zukunftsfähiger zu gestalten.

## Background

In order to increase the quality and efficiency of vocational (=post-graduate) training in general practice (=GP), competence centers for vocational training (KW) were founded in 2017 [1]. Based on the experience of the Verbundweiterbildung^plus^ as the first project of its kind in Germany (2009-2017) [2], [3], the KW in Baden-Württemberg (KWBW) offers the KWBW-Verbundweiterbildung^plus®^, a concomitant program that accompanies the training post [https://www.weiterbildung-allgemeinmedizin.de/]. An essential component is the seminar program designed for post-graduate trainees in GP (=GP-trainees) [4]. In 2019, the KWBW conducted 39 seminar days, in which a total of 379 GP-trainees participated.

While digital formats were already used in medical education before the Covid-19 pandemic [5], [6], they have rarely been used for vocational training in Germany. Since the regulations for protection against SARS-CoV-2 [7] were issued, the KWBW has converted the seminars that had already been planned in presence into digital formats in only four weeks. The project implementation was based on a modified Kern cycle [8]. In accordance with the requirements and objectives (steps 1-3 according to Kern), it was decided to hold up the standards of high quality in content and educations while maintaining the interactive character in the online format. A synchronous teaching format in the form of webinars was chosen as teaching method (step 4 according to Kern). For a better understanding of the content, the possibility of more intensive work during the seminars and to promote in-depth learning [9], preparatory and follow-up tasks were specified, based on the online inverted classroom model (oICM) [10]. This is a variant of the flipped classroom method [11]. To stimulate self-reflection, the preparatory tasks were carried out with a portfolio, the competence-based curriculum for general practice [12]. For the implementation (step 5 according to Kern), the technical aspects were considered as threats when implementing the E-EST. For this purpose, participants were requested to use a cable-based Internet connection, a headset and a software-based application (instead of a browser-based one). The evaluation (step 6 according to Kern) was carried out with a multicenter newly developed and pre-piloted questionnaire. For the participants it contained six questions on sociodemographic data and 22 questions on the format of e-learning. For the lecturers it contained six sociodemographic questions and 29 questions on e-learning.

The aim of this study was to evaluate the acceptance of digital teaching formats and to explore the advantages and disadvantages compared to traditional face-to-face teachings from the trainee’s (=GP-trainees) as well as trainers’ (=lecturers) perspective.

## Results and discussion

**Figure 1:**
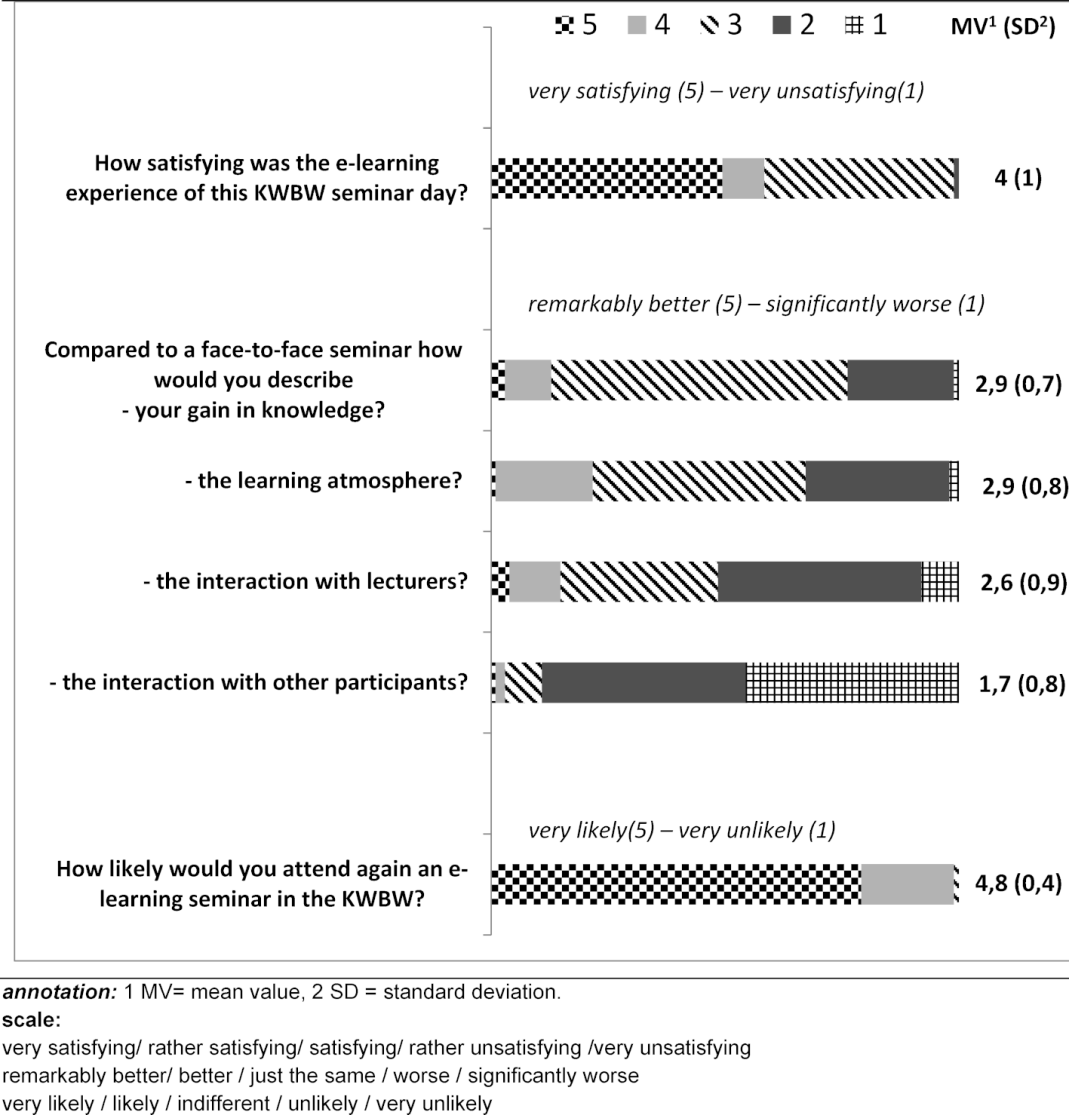
Evaluation of e-learning in the KWBW-Verbundweiterbildung^plus^ by post-graduate trainees in general practice (n=101)

From 23.04.2020 to 09.05.2020 five seminar days were conducted online (offer for 125 GP-trainees). n=104 attended. A total of 101 GP-trainees (response rate 97.1%) and all lecturers (n=11) who were not employees of the KWBW participated. Table 1 (Tab. 1) depicts sociodemographic data. 28.7% (n=29) of GP-trainees and 45% (n=5) of the lecturers had already experienced e-learning. 52% (n=53) of the GP-trainees and 55% (n=6) of the lecturers have changed their attitude towards e-learning (all comments in the free text sections were positive changes in attitude). The results of the evaluation from the GP-trainees’ point of view are shown in figure 1 (Fig. 1). Nine lecturers (82%) were satisfied or very satisfied with the idea of e-learning, all of them would like to be involved in a digital format again. 55% (n=6) assessed the learning success for GP-trainees to be worse, 45% (n=5) considered it comparable to classical teaching formats. All lecturers felt that the interaction with the GP-trainees was worse (n=7) or significantly worse (n=4). Advantages and disadvantages of e-learning from the trainees’ and trainers’ perspective are shown in table 2 (Tab. 2) and table 3 (Tab. 3). 

In this short report we show for the first time that the conversion of an education program for GP-trainees into a digital format is possible at short notice and can be seen as an opportunity by the learners. Previous studies of the acceptance of e-learning in continuing medical education, reported a reserved basic attitude [13]. This seems to have changed positively with the increasing spread of electronic media [14], [15], which our results confirm. Half of the GP-trainees stated that their attitude towards e-learning had changed positively through participation. The main reasons cited were that it had exceeded existing expectations (n=13) and was a good alternative in the current situation (n=5). The limited opportunities for interaction with the lecturers and with each other were critically assessed. The explicit goal of the KW is to contribute not only to the transfer of knowledge, but also to the networking among GP-trainees and their professional identity in GP [2]. However, the limited exchange of information has also been described by others [16], e.g. students would also like to have more opportunities for interaction in e-learning [17]. Lecturers evaluated the e-learning experience surprisingly positive, but scrutinized whether the increase in learning is comparable to a classroom event. Qualitative studies with lecturers are necessary with regard to the justification of this position as well as possible changes in attitude. 

The high acceptance of the flipped classroom method shows that the integration of activating elements can be successful. The preparatory tasks were considered to be helpful by n=83 (82%). The reasons given were a positive effect on learning success (n=30), a better understanding of content (n=25) and a comparable level of knowledge of the ÄiW (n=6). This method has rarely been evaluated in vocational training in Germany [5]. Studies with medical students show that it can contribute positively to motivation and commitment [18]. In particular, it can help to promote deep learning [19]. These effects are not only limited to e-learning [20], therefore the positive attitude of the ÄiW described by us can also motivate a broad implementation of the flipped classroom in face-to-face seminars.

A strength of this study is the high number of responses. Approximately one fifth of the places stayed vacant (comparable to participation in face-to-face seminars). We do not know whether professional or family obligations during the pandemic or the format itself (e.g. insufficient access or lack of inner readiness) influenced the registration. Changes in attitude can only be rudimentarily presented in a questionnaire, for a more detailed exploration a qualitative follow-up study is necessary. 

Despite all the opportunities offered by the digital possibilities, it is important to maintain the regional personal reference during the seminar days. During medical school and the clinical training of vocational trainees there is interdisciplinary and social interaction with colleagues on a regular basis. These opportunities are not available in general practice. This gap had been closed by our face-to-face seminars [2]. E-learning can contribute to a better compatibility of education and family tasks, especially for GP-trainees in rural regions. It can hereby further increase the attractiveness of general practice. The results of our work motivate us to implement e-learning beyond the Covid-19 pandemic as a supplement at the KWBW. Further studies on e-learning in vocational training and continuing medical education are needed with an emphasize on how the social interaction of colleagues can be improved parallelly to a continuous increase in learning.

## Competing interests

The authors declare that they have no competing interests. 

## Figures and Tables

**Table 1:**
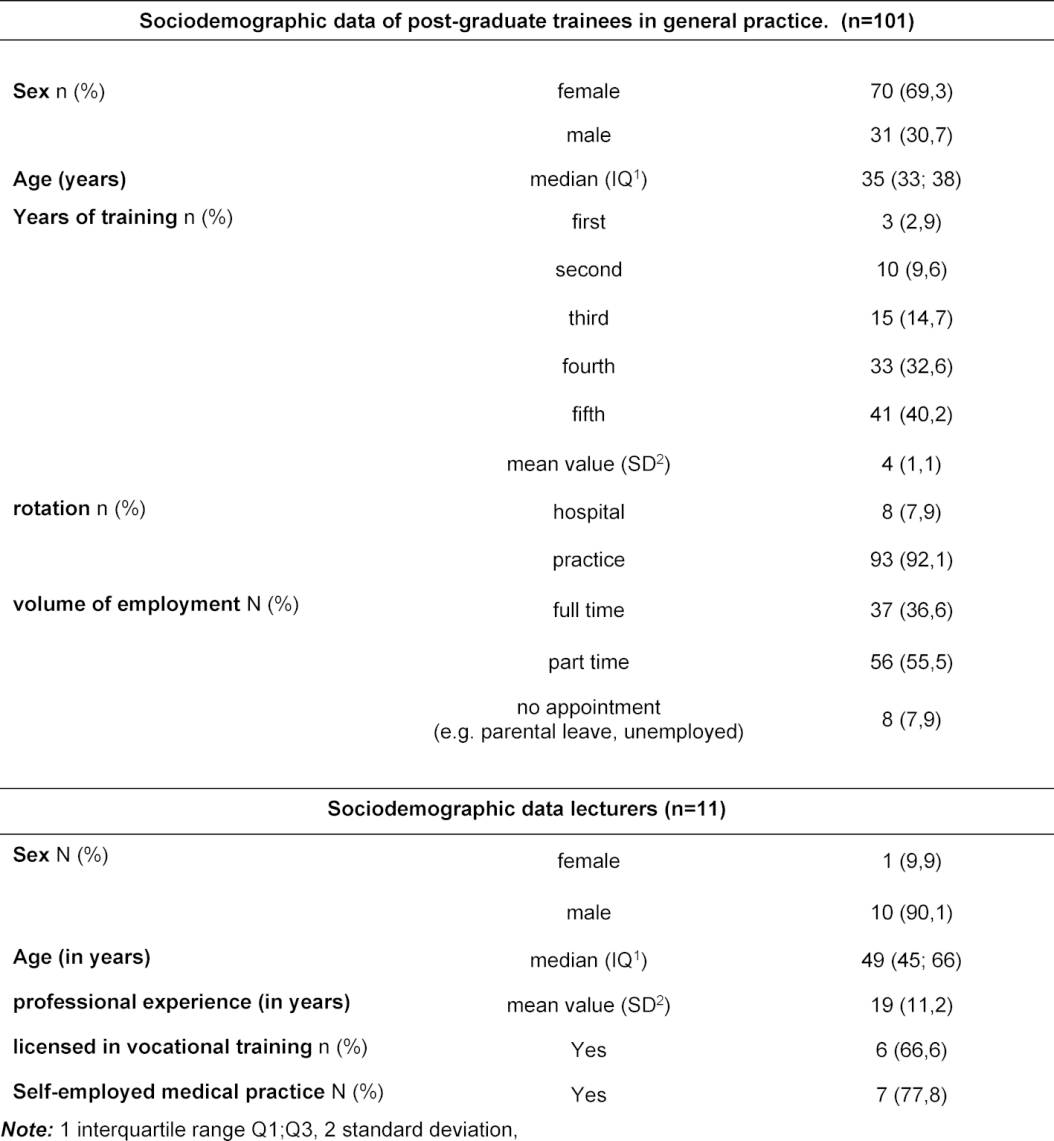
Sociodemographic data of post-graduate trainees in General Practice and lectures

**Table 2:**
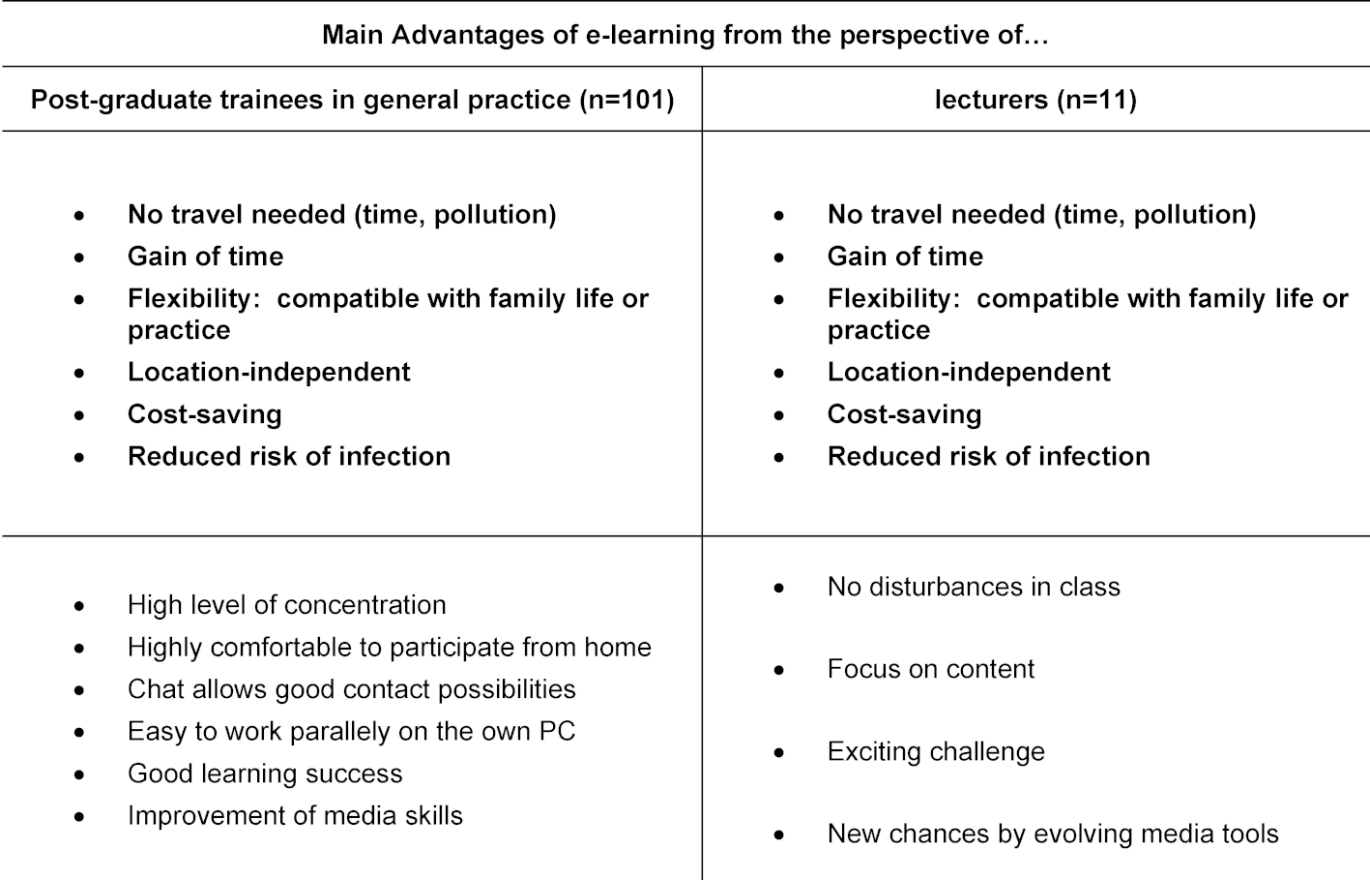
Advantages of E-Learning from the perspectives of post-graduate trainees in general practice and their lecturers

**Table 3:**
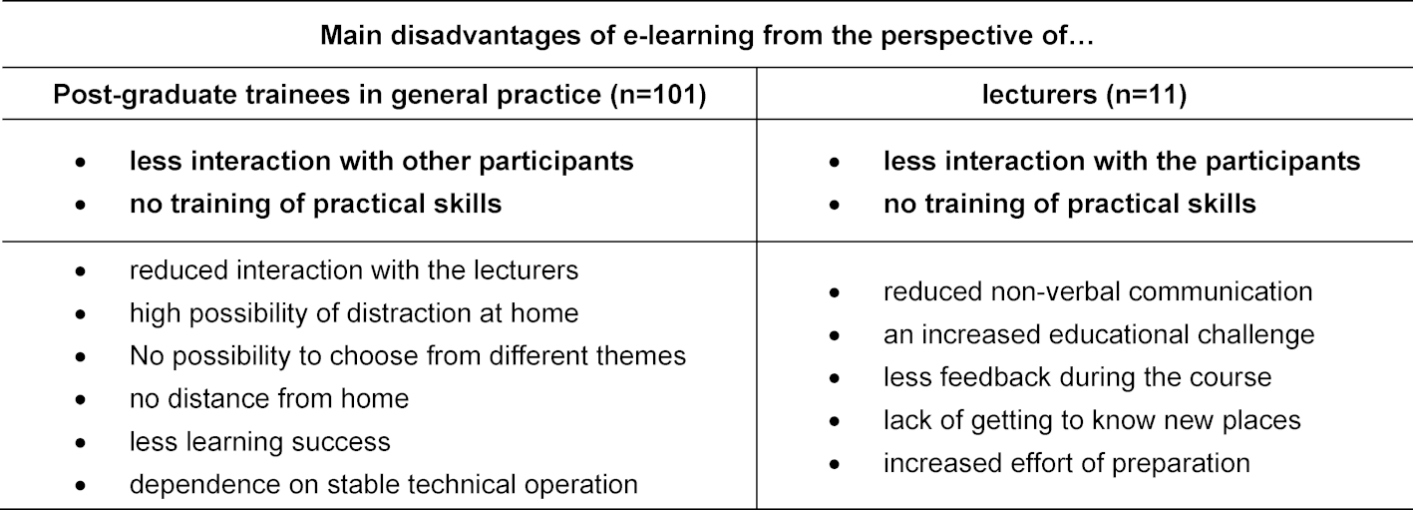
Disadvantages of E-Learning from the perspectives of post-graduate trainees in general practice and their lecturers
